# The Relationship between Cognitive Status and Retained Activity Participation among Community-Dwelling Older Adults

**DOI:** 10.3390/ejihpe12040029

**Published:** 2022-03-29

**Authors:** Fatemeh Adelirad, Maryam Moghaddam Salimi, Iman Dianat, Mohammad Asghari-Jafarabadi, Vijay Kumar Chattu, Hamid Allahverdipour

**Affiliations:** 1 Department of Health Education and Promotion, Tabriz University of Medical Sciences, Tabriz 14711, Iran; adelirad@tbzmed.ac.ir; 2 Department of Physiotherapy, Tabriz University of Medical Sciences, Tabriz 14711, Iran; moghadamm@tbzmed.ac.ir; 3 Department of Occupational Health, Tabriz University of Medical Sciences, Tabriz 14711, Iran; dianati@tbzmed.ac.ir; 4 Department of Statistics and Epidemiology, Zanjan University of Medical Sciences, Zanjan 45154, Iran; asgharimo@tbzmed.ac.ir; 5 Center for the Development of Interdisciplinary Research in Islamic Science and Health Science, Tabriz University of Medical Sciences, Tabriz 14711, Iran; 6 School of Public Health, University of Alberta, Edmonton, AB T6G 1C9, Canada; 7 Center for Transdisciplinary Research, Saveetha Institute of Medical and Technological Sciences, Saveetha University, Chennai 600077, India; 8 Department of Community Medicine, Faculty of Medicine, Datta Meghe Institute of Medical Sciences, Wardha 442107, India; 9 Research Center of Psychiatry and Behavioral Sciences, Tabriz University of Medical Sciences, Tabriz 14711, Iran

**Keywords:** aged, activity card sort, attention, balance, executive function, memory

## Abstract

Identifying retained activity participation to old age can improve age-related changes in balance and cognition function. Subjects ≥ 60 years were enrolled in this study. Balance and Cognitive function include working memory, executive function, and sustained and divided attention was evaluated with “Fullerton advanced balance”, “n-back”, “Wisconsin card sort”, “sustain and divided attention test”, respectively. In addition, retained activity participation was measured using the Activity Card Sort questionnaire. The univariate and multivariate regression analyses of different domains of retained activity participation were used as independent variables, including instrumental activity, low-effort leisure, high-effort leisure, and social activity on balance and specific domains of cognition. Seventy-seven subjects (65.3 ± 4.4 years, 61% female) were included. About 47% of older adults had a college education, 32.3% had a diploma, and 20.7% had elementary–middle education. These results show that retained instrumental activity had a relationship with working memory (*β* = 0.079, *p* < 0.05). In addition, we found that retained high-effort leisure activity can increase balance, divided attention, and executive function score (*β* = 0.1, *β* = 0.05, *β* = 0.02, *p* < 0.05). Moreover, there was a positive relationship between retained low-effort activity and sustained attention (*β* = 0.08, *p* < 0.05). In addition, the coefficient of determination (R^2^) for balance, working memory, executive function, sustained, and divided attention were 0.45, 0.25, 0.13, 0.11 and 0.18, respectively. The study suggests that retained activity participation types may have various effects on balance and some selective cognitive components in older people.

## 1. Introduction

Cognitive and physical function are two important aspects of aging health, and maintaining both at a high level is critical for improving autonomy and being successful in aging [1,2]. Rowe and Kahn’s postulated that successful aging depends on avoiding disease and disability, high cognitive and physical functioning, and remaining engaged in the community and society [3,4].

Additionally, about 28–35% of people aged 65 and over fall each year, increasing to 32–42% for those over 70 years [5]. Moreover, it is still not clear what types of training lead to improvements in balance and prevent falls. Besides, there are controversial shreds of evidence about the effectiveness of some types of exercise training. For example, there is evidence that Wii Fit training (a new technological approach of balance training) is a useful approach for reducing the risk of falls and improving the dynamic balance [6]. In contrast, other relevant evidence reported that Wii Fit is ineffective in improving dynamic balance among the community-dwelling elderly [7]. Therefore, there has been increasing interest in identifying factors, activities, and strategies that might keep or prevent cognitive and physical function and slow down its decline in recent years.

On the other hand, older adults commonly experience cognitive decline, and they are at increased risk of the onset of cognitive impairments such as dementia [8]. The most common changes in cognition are declines in cognitive tasks that require transforming information to make a decision [9]. For example, the most common important age-related changes in cognition appear in the frontal cortex, such as changes in executive function, attention, and working memory [10,11]. Thus, focusing on working memory, executive functioning, and sustained and divided attention would be critical. Attention is the heart of cognitive aging and as a fundamental cognitive process, it may also act as a mediator of decline in other cognitive abilities, such as memory and reasoning [8]. The age-related attention decline frequently appears in the efficiency of sustained and divided attention performance [9,10]. The ability to maintain the focus of attention on a task over time is known as sustained attention, such as engaging in problem-solving in a noisy environment [11]. Likewise, divided attention is defined as the ability to focus on multiple tasks simultaneously, such as talking with someone while preparing a meal [12]. This type of attention is likely to be the earlier domain of attention control, which is influenced by Alzheimer’s disease [13]. Behavioral research suggests that sustained and divided decline is apparent as perceptual demands increase, thereby increasing task difficulty and decreasing the quality of almost every area of older adults’ life, such as driving, maintaining balance, and financial managing [14,15,16].

Furthermore, neuroimaging studies indicate that age-related reduction in attention occurs, but that age-related changes in the cortical synapse of the dorsolateral prefrontal cortex affect working memory and executive function. [15,17,18]. The executive cognitive function involves decision making, problem-solving, planning and sequencing of responses, and multitasking, each of these domains of executive cognitive function declines with the aging process [17]. Additionally, working memory is a multidimensional cognitive construct hypothesized as the fundamental source of age-related deficits in various cognitive tasks, including long-term memory, language, problem-solving, and decision making [9]. Decline in executive function and working memory observed with ageing has been associated with significant limitations of functionality, independent living, mental math and consequently, a decline in quality of life and successful aging [19,20,21]. In a preventive manner, there is some evidence that age-related changes in balance, divided attention, sustained attention, executive function, and working memory can be improved by traditional Srichiangmai dance [22], aerobic exercises [16], video games [14], brain training games [23], and mind–body exercises [24].

At the same time, there is some evidence that shows age-related deficits in executive function are not improved by cognitive-motor training [25], and also working memory is not enhanced by resistance exercise training [26].

Many medical and sports sciences experts believe that being active and regular physical activity during middle and elderly age is associated with better health in later life [27,28]. Retained active engagement, especially in instrumental, social, and leisure activities during middle age to later life, has been found to slow the onset of some health risks and reduce the probability of disease-related disability. For instance, in a five-year follow-up study of 6345 older adults, it was found that engaging in high levels of leisure activity helped reduce the risk of cognitive impairment by 41%, even after controlling covariates [29]. Furthermore, we observed a wide range of leisure activities (such as swimming and walking) that have different relationships with balance. For example, a long-term follow-up study found that swimming training can improve balance function and reduce the risk of falls [30]. In contrast, another study shows that long-term walking (often walking is the only leisure-time physical activity for older adults), does not protect from falls [31].

Generally, regarding activity and participation, the World Health Organization highlights the importance of a tool like the Activity Card Sort (ACS) that allows researchers and clinicians to further understand participation issues and monitor changes over time [32]. ACS is one such comprehensive tool that provides useful information on the individuals’ participation patterns in instrumental, social, high-effort leisure, and low-effort leisure activity [33]. This instrument measures the level of perceived participation via picture cards depicting the daily activity. The assessment requires the older adult to sort the pictures according to their past (before 60 years) and current levels of participation for each activity and calculate retained activity participation. In addition, the ACS was used as an assessment tool to measure the effectiveness of interventions in populations with different health conditions, such as cognitive oriented strategy training in post-stroke older adults [34]; and cognitive self-management in older people with breast cancer and cognitive impairment [35].

To the best of our knowledge, there have been no studies that have investigated the relationship between domains of retained activity participation (instrumental, leisure, and social activity) with physical function (including balance (as one domain of physical activity) and cognition (including executive function, working memory, sustained and divided attention) in older adults. Studies that have examined the potential connection between older adults’ activity participation with motor and cognitive skills limited with contradictory results. In fact, we want to identify “Which of the different domains of retained activity participation are associated with which of the selected components of cognition and physical function?” If the association is confirmed, these findings may lead to numerous optimal guidelines and recommendations that maximize the neuroplasticity properties of the selected cognitive domains and balance, which can be investigated in longitudinal research.

This study aimed to (1) investigate the association between demographic characteristics with balance, executive function, working memory, and sustained and divided attention; (2) examine the correlations between four domains of retained activity participation with balance, executive function, working memory, and sustained and divided attention; and (3) to assess the relationship between four domains of retained activity participation, health status with balance, executive function, working memory, and sustained and divided attention.

## 2. Materials and Methods

### 2.1. Participants and Procedures

This cross-sectional study was conducted on 77 older adults in Tabriz from August 2020 to January 2021. In this study, the sample size was estimated by the following formula [36]:

Total sample size=N=[(Zα+Zβ)/C]2+3=51

where ‘*N*’ is the required sample size, and is the normal deviate for the two-tailed alternative hypothesis at a level of significance. The level of significance (*α*) is set at 5%, and thus the standard normal deviate for *α* = *Zα* = 1.9600 equals. *β* is the Type II error rate and set at 80%, thus the standard normal deviate for *β* = *Zβ* = −0.8416. A correlational study in the UK estimated that the correlation coefficient between executive function and leisure-physical activity was 0.2. Therefore, according to the formula, the required sample size was determined to be 51. There should be adequate power since the actual sample size (77) was far more than the required sample size. Participants were recruited by using a convenience sampling method from four existing geriatric health centers in Tabriz, Iran. All the eligible older adults who were admitted satisfied the inclusion criteria. The inclusion criteria for the adults age 60 years or older were: the ability to walk independently, no visual or auditory impairment, no history of cognitive impairment (Mini-Mental status Examination > 23), and no history of neurological or psychiatric disorders. Among the invited participants, 77 men and women completed the informed consent, written questionnaire, and computer-based test. This study was approved by the Ethics Committee in Tabriz University of Medical Sciences (Ethics Code: IR.TBZMED.REC.1398.829).

### 2.2. Measurement

#### 2.2.1. Demographic Characteristics and Health Status

Demographics information and health status data were age, gender (male/female), marital status (single, married), literacy level (elementary to Middle school, diploma, College education), Job (retired, unemployed),) hypertension (Yes/No), diabetes (Yes/No), hyperlipidemia (Yes/No), number of current diseases (0, 1, ≥2); diseases in this study include high blood pressure, high blood fats and diabetes, so number of diseases were between 0–3), medication use (0–2, 3≤) and history of falling (Yes/No).

#### 2.2.2. Balance

The Fullerton Advanced Balance (FAB) Scale was developed by “Rose DJ” to assess postural control in higher functioning older adults [37]. It consists of ten items that require static and dynamic postural control, sensory reception and integration, and feedforward/feedback postural control. The FAB scale consists of ten items: “balancing with feet together and closing eyes”, “forward reach”, “turn 360 degrees”, “stepping up, onto and over a six-inch bench”, “tandem walk”, “standing on one leg”, “standing on foam with closed eyes”, “two-footed jump”, “walking with head turns”, and the performance in each of the ten individual test items is scored using a five-point ordinal scale (0–4) with a maximum score of 40 points possible, indicating “postural reaction” [37]. The Persian FAB scale has shown good to excellent test–retest reliability (reliability (Cohen’s Kappa = 0.6–1) as well as intra- (0.9–1.0) and inter-rater reliability (0.91–0.95). Internal consistency was acceptable (Cronbach *α* = 0.83–0.84) for both phases [38].

#### 2.2.3. Executive Function

Among various neuropsychological tests used to assess executive function, the Wisconsin Card Sorting Test (WCST) is one of the most well-known and used in clinical and research practice [39]. The WCST provides relevant information about numerous aspects of executive function. The test consists of matching test cards one by one with stimulus cards, following a rule that the participants must deduce. Therefore, we used 64 test cards which had to be associated with four stimulus cards (1—one red triangle; 2—two green stars; 3—three yellow crosses; and 4—four blue circles). In the WCST, participants were presented with a deck of cards to sort according to one of three properties (color, shape, or number of shapes); next, the participant received positive or negative feedback on their performance through the words “right” or “wrong” presented on the screen. We used a parameter to evaluate performance by the total number of correct answers and the reaction time. One response card per trial appears in the middle of the screen, and the participant has to try to match it to one of the four stimuli cards [40,41]. Previously published research which assessed the psychometric properties of TWCST has shown good to excellent reliability using Cronbach’s alpha coefficient (alpha = 0.9); Pearson’s correlations between the WCST indexes and the MMSE showed that the following indexes achieved sufficient convergent validity (r between 0.2–0.5) [42]. Additionally, Cronbach’s alpha (alpha = 0.8) and split-half coefficients of the computerized version showed suitable reliability in Iranian subjects [43]. In this study, the correct number and reaction time of the test were measured. In Iran, this computer-based program is made by the Sina Research Institute of Behavioral-Cognitive Sciences [43].

#### 2.2.4. Working Memory

Gevins and Cutillo utilized the N-back task to evaluate working memory [44]. In this test, participants are shown a series of numbers and are instructed to press a button when the current stimulus is the same as the item presented in n-positions. In this study, one-back versions of the task were used; therefore, the participant must compare each number with the previous number. If the current number is similar to the previous one, the ”?” key is pressed, otherwise the “Z” key is pressed. Item lists included 48 target and non-target items. Each item was presented for 1500 millisecond(ms). In this study, the correct number and reaction time of the test were measured. In Iran, this program is made by the Sina Research Institute of Behavioral-Cognitive Sciences using a simulated approach to explain working memory and its components [45].

#### 2.2.5. Sustained and Divided Attention

This test was made by Khodadadi et al., 2012, to measure sustained and divided attention in different age groups [46]. In the first step for sustained attention, different shapes appear on the computer screen one after the other and the participant must look at them carefully. Whenever the participant sees any shapes of candles and circles, the participant must quickly click the space key. In the second step for divided attention, the participants have to look at them carefully each time at two shapes appearing simultaneously on the computer screen. Whenever the circle is on the right side of the computer screen, or the candle is on the left side of the screen, the participant must quickly click on the “?” and “z” keys, respectively. Moreover, if both shapes are presented on the computer screen at the same time and the circle in on the right side and candle is on the left side, participants must quickly press the “?” and “z” key at the same time. In this study, the correct number and reaction time of the test were measured. In Iran, this software is made by the Sina Research Institute of Behavioral-Cognitive Sciences using a simulated approach to explain sustained and divided attention and its components. The validity of this tool has been reviewed and approved by the experts [46]. Additionally, Zare et al. reported the Cronbach’s alpha coefficient of this questionnaire as 0.8 for sustained attention and 0.9 for divided attention. In the evaluation of validity between inconsistent response in the Stroop test and sustained attention, a correlation coefficient of 0.390 was obtained [47].

#### 2.2.6. Retained Activity Participation

The Activity Card Sort (ACS) is a tool used to measure an individual’s participation level in instrumental, leisure, and social activities based on an interview [33]. This instrument measures the level of perceived participation via picture cards depicting daily activities. The ACS has four domains, including instrumental activity (e.g., driving, paying bills, childcare), low-physical-demand leisure (e.g., puzzles, reading the newspaper, watching TV), high-physical-demand leisure (e.g., swimming, walking, table tennis), and social activity (e.g., volunteer work, visiting with friends, traveling). The subject sorts the card into four categories (‘I have done as an adult’, ‘given up’, ‘do less’, ‘do now’). The ACS provides an activity level (score range: 0–100), which is the percentage of activity a person is currently engaged in, compared to those they were involved in before 60 years [32,33,48]. It has been translated into several languages and made appropriate to several countries, such as Hong Kong [49], Australia [50] and Spain [51] and the United Kingdom [52]. The ACS-Persian version presents appropriate psychometric characteristics, with high internal consistency Cronbach’s alpha values of 0.8 for instrumental activity, 0.7 for low-physical-demand activity, 0.8 for high-physical-demand activity, and 0.7 for social activity. The test–retest correlation was 0.7 and a good level of test-retest reliability [32].

### 2.3. Statistical Analyses

All analyses were performed using IBM SPSS Statistics (version 24 IBM Corp, Armonk, NY, USA). Data were presented as frequency (percentage) for categorical variables and as mean (SD) for numeric variables. The distribution of data was normal according to the Kolmogorov–Smirnov test. We used the Pearson correlation coefficient to determine the correlation between variables. In cognitive research, speed and accuracy are two important aspects of performance. Therefore, for all cognitive tests of this study, the number of correct answers of each test was divided by the reaction time of the same test ((correct answers (n))/(reaction time (s))). Moreover, retained activity participation in each domain was calculated by dividing the current activity participation of each domain by the previous activity participation of each domain (before 60 years) A series of univariate general linear models were used to assess the relationship between demographic-health variables and the level of retained activity participation including instrumental activity, low-effort leisure, high-effort leisure, and social activity as independent variables, with balance and specific domains of cognition including working memory, executive function, and sustained and divided attention. This model was run in both univariate and multivariate analyses, and in the multivariate analysis, the effect of possible confounders was adjusted. In the models mentioned above, unadjusted and adjusted regression coefficients and their 95% CIs were estimated for univariate and multivariate models, respectively. Qualitative variables were entered into the models as indicators. In all analyses, *p* < 0.05 was considered significant.

## 3. Results

An overview of sample characteristics is given in Table 1. The study population included 30 men and 47 women with a mean ± SD age of 65.3 ± 4.4 yrs. Table 1 also shows the balance and cognitive components according to background variables. Correlation analysis on these data showed a significant relationship between participants’ age and their balance scores (r = −0.2; *p* < 0.05). Furthermore, another significant relationship was found between participants’ age and their sustained attention scores (r = −0.2; *p* < 0.05), although was not found between age and balance with other cognition components. As expected, men had significantly lower mean scores in working memory (r = 0.2; *p* < 0.05) and balance than women (r = −0.2, *p* < 0.05). According to the results, there was a significant relationship between the number of diseases with mean score working memory (r = −0.2, *p* < 0.05), and balance (r = −0.2, *p* < 0.05). In other words, the participants who had no history of disease had better working memory and balance than those who had at least one disease. In addition, correlation analysis of the data showed a significant difference between employment types and balance; between retired and working memory; and also between hyperlipidemia and executive function (r = 0.2, *p* < 0.05; r = 0.2, *p* < 0.05; r = 0.2, *p* < 0.05, respectively).

Table 2 illustrates the Pearson coefficient of correlations to examine the correlation between balance, working memory, executive function, sustained attention and divided attention, with various domains of activity participation. As shown in Table 2, a simple significant correlation was found between balance function with instrumental activity (r = 0.2, *p* < 0.05) and high-effort leisure activity (r = 0.3, *p* < 0.05). Moreover, the analysis showed a significant correlation between working memory with high-effort leisure activity and low-effort leisure activity (r = 0.05, *p* < 0.05; r = 0.1, *p* < 0.05). Furthermore, there was a significant correlation between executive function and social activity (r = 0.1, *p* < 0.05). Lastly, a simple significant correlation was found between high-effort leisure activity and divided attention (r = 0.2, *p* < 0.05).

The results of the univariate analysis are shown in Table 3 and Table 4. In Table 3, the univariate general linear model analysis showed an association between sustained attention and number of diseases (*β* = 1.8, *p* < 0.05) and low-effort leisure activity (*β* = 0.08, *p* < 0.05). Eleven percent (11%) of the sustained attention was explained by retained activity participants and the demographic variables. In other words, those who have one disease (compared to at least two diseases), or those who have more low-effort leisure activity, have better sustained attention scores. Results revealed that those who have elementary education had a lower (0.8) divided attention score compared to those who have college education (*p* < 0.05). Moreover, our findings indicated that those who have participated in high-effort leisure activity have higher scores in divided attention (*β* = 0.05, *p* < 0.05), and those who have hyperlipidemia have lower scores in divided attention (*β* = −2.3, *p* < 0.05). The R^2^ of the test was 0.18, indicating that 18% of the divided attention was explained by retained activity participants and the demographic variables.

## 4. Discussion

Our cross-sectional study showed that older adult engagement with a high-effort leisure activity is linked to having a higher score in divided attention, executive function, and balance performance. In addition, the study revealed a positive association between engaging with low-effort leisure activity and performance in sustained attention. Furthermore, in line with these results, older adults with higher engagement in instrumental activity had higher working memory function. Finally, these findings suggested that participation in a high-effort leisure activity may be an important factor for improving cognitive and balance function. We now provide potential explanations for these findings and discuss how these findings can be applied in clinical practice.

Throughout this research, it was observed that instrumental activity was associated with higher working memory function. Our current study extends a previous study that revealed that greater participation in instrumental activity was associated with higher working memory function [53]. In Iran, Nourbakhsh et al. reached a similar finding, meaning that the cognition of older adults was dependent on instrumental activity [54]. In another study Toth et al. found the relationship between working memory and ‘paying bills’ as one of the instrumental activities of daily living [55]. Additionally, Reppermund et al. suggest that difficulties in instrumental activity, especially those with a higher demand on cognitive capacities such as medication responsibility, shopping, and finance-handling, are associated with a deficit in cognitive function [56]. A possible reason is that some instrumental activity such as “calculating the total bill of groceries in e-shopping (mental math)” may reinforce the ability to hold onto and use specific information for a certain amount of time (working memory capacity) through the shopping. Based on the results, instrumental activity such as (such as paying the bills, shopping) can be used to reframe and improve intervention strategies for improving working memory and maintaining performance in daily activities.

This research observed that engaging in high-effort leisure activity improved divided attention, balance, and executive function. It was also reported that those with a higher score of high-effort leisure activity (such as swimming and walking) had higher balance function [57]. These results are supported by study of Resende et al. that investigated the effects of a hydrotherapy program on balance, and the risk of falls in elderly women. This study found that hydrotherapy promoted the elderly women’s balance and reduced the risk of falls [58]. This is likely to mean that physical activities such as running or swimming may help older adults to enhance their balance-related physical properties, such as ankle and knee joint proprioception [27].

Additionally, increased balance function in those with high-effort leisure activity may improve musculoskeletal function, faster movement, more range of motion, and sensorimotor function [58,59]. Furthermore, engaging in high-effort leisure activities throughout life also improves brain motor strategies to maintain balance performance and cognitive abilities [60]. It is important to point out that because tennis and swimming require alertness and tactical thinking, it may increase neurogenesis and promote brain development [61,62,63]. Regarding the relationship between high-effort leisure activity and balance status, the high-effort activity interventions need to consider promoting both the cognitive and balance function. In fact, research is needed to determine whether changes in high-effort leisure activity can causally affect the falling trajectory.

The results of this study suggest that the high-effort leisure activities may improve executive function. Several meta-analyses support this by showing a low-to-moderate effect size on the improvement of cognitive aspects, especially executive function, after aerobic exercise sessions [64,65]. In line with our findings, one cross-sectional study demonstrated the bidirectional relationship between physical activity and executive function. Those with poor executive function showed decreases in their rates of participation in physical activity, and older adults who engaged in sports tended to retain high levels of executive function over time [66]. From a basic research perspective, neuronal level, enhancing synapses, increasing blood flow, activation of the prefrontal cortex, and vascularization are potential mechanisms of high-effort activity that may improve executive function [67,68]. Interestingly, this result may be related to “Temporal Self-Regulation Theory: A Model for Individual Health Behavior” (Hall and Fong, 2007), which suggests that executive functioning will be essential for the enactment of behaviors like physical activity [69]. On the other hand, these findings may explain potential interrelationships between motor skill performance and executive functions via inhibitory control of movements [70]. It is important that future research explores the impact of exercise training on cognitive flexibility. Furthermore, our results provide additional support for the notion that high effort leisure activity (e.g., walking, yoga, driving) may enhance divided attentional control mechanisms in older adults.

Only a few studies were found on divided attention training. Most of them examined the effectiveness of computer-based games with contradictory results that may be due to the amount of video game training provided [71,72]. Consistent with our findings, Angevaren et al., in a systematic review study, found that aerobic activity is beneficial to attention function in older people [73]. Some main biological mechanisms that appear to underlie exercise-induced cognitive improvement among older adults are increased serum levels of brain-derived neurotropic and insulin-like growth factor type one [74]. Divided-attention is widely involved in high-effort activities such as driving activities. For example, drivers must maintain attention on a stable trajectory while paying attention to traffic, so this simultaneous attention to different aspects of high-effort leisure activity may be a potential reason for this association. However, regarding the importance of divided attention, especially in dual tasks, it is suggested that further studies be conducted on the design of dual-task-high-effort leisure activity interventions to evaluate the effect of divided attention. In addition, our findings indicated that only low-effort leisure activity (e.g., listening or playing a musical instrument, word puzzle, reading books) has an association with sustained attention. This association between low-effort leisure activity and sustained attention was also reported by Cloutier et al. [75], Brooker et al. [76], Wessel et al. [77] and Chen et al. [78], about the relationship between attention and music listening and playing, word puzzles, chess, and reading. At the same time, other studies are contrary to our findings of music listening and music playing [79,80]. These diverse findings of the effect of music or chess may be due to the frequency and period of intervention administration, the target population, and different chronic conditions. The possible mechanism of the influence of music on sustained attention maybe through the speed of action. Practicing music may result in an increased amount of myelin and a fatty substance that covers axons via the myelination process, resulting in faster and more efficient message transmission between neurons [67]. In addition, regularly playing chess may activate the parietal lobes of the brain for possible control of attention and spatial orientation [81]. However, it can be argued that the ability to maintain a relatively stable state of attention continuously is essential for complex cognitive activities such as chess, word puzzles, and reading, which is the definition of sustained attention [82]. Doing this low-effort activity for a long time from middle age to old age can probably positively affect attention.

We observed that employed older adults were likely to have a better balance in the relationship between cognition and balance. This result is contrary to Sharif et al.’s findings [83], while consistent with the findings of Tabatabaei et al. [84] and Taekyoung Kim [85], which reported that retired older adults had significantly lower fall-risk scores. Employment may have given older adults an advantage in muscle reserve capacity, critical thinking ability, and better maintenance of balance [86]. Finally, our findings showed that literacy level might improve attentional function. One possible pathway may be that the involvement in learning activities may improve cognitive function through life activities and help to maintain brain reserve [87]. Some probable ways to optimize cognitive reserve is through staying active in the community and study. A practical suggestion to older adults would be to engage in more social interaction with friends and family, volunteer in charities, and continue their education.

It should be noted that we used the Bonferroni correction for multiple comparisons (a multiplicity adjusted *p*-value) to estimate the probable error rate and control it at an appropriate level (*p*-value < 0.05). Accordingly, some significant relationships were lost after calculating Alfa error accumulation in this study, which may be due to the interrelationship effect of the measured variables.

The strengths and limitations of the study must be acknowledged. To our knowledge, this is the first study to investigate balance, executive function, working memory, sustained attention, and divided attention with retained activity participation in older adults. Secondly, the tests used to measure activity participation in this study allowed analysis of four activity participation types: industrial/social/high-effort/low-effort activities. Thirdly, cognition was assessed with different tests that allowed evaluation of specific cognitive domains. Finally, potential factors that could influence the association between balance and cognitive functions with activity participation, such as intelligence and depression, were considered in the inclusion criteria, and the results were not affected by them.

### Limitations

However, we recognize our study limitations in spite of the strengths discussed above. Firstly, the number of the subjects was not very large. Secondly, there might be a selection bias among subjects because the sample was composed of participants with higher education levels and who had no history of stroke, so the results could not be generalized to all older Iranian adults living in the community. Finally, despite the effort of adjusting for a number of confounders such as demographic characteristics, we could not completely eliminate the risk of confounding bias such as medication types; this may have affected the results, as some medication is known to interact with cognition.

## 5. Conclusions

In conclusion, this study highlighted the importance of retained activity participation, especially high-effort leisure activities, as part of a healthy lifestyle that may protect against cognitive impairment and imbalance. These findings could lead to draft practice guidelines that promote each cognition component and balancing function to the greatest extent possible. Future research should compare the types of exercise involvement to the cognitive components once the subjects have been followed up in a longitudinal study to provide conclusive evidence supporting these findings.

## Figures and Tables

**Table 1:** Correlations and Descriptive Statistics: demographic health information, balance, working memory, executive function, and sustained and divided attention.

Variable		Mean ± SD or Frequency (%)	Balance	Working Memory	Executive Function	Sustained Attention	Divided Attention
Age (Year)		65.3 ± 4.7	30.5 ± 4.7 *	17.5 ± 12.9	10.1 ± 3.8	32.6 ± 2.7 *	16.5 ± 4.0
Gender	Male	30(39.0)	32.1 ± 3.50 *	21.1 ± 19.4 *	10.2 ± 4.2	32.3 ± 2.4	16.7 ± 3.0
	Female	47(61.0)	29.5 ± 5.2	15.2 ± 4.7	10.0 ± 3.6	3 2.8 ± 2.8	16.4 ± 4.5
Education level	Elementary–Middle	16(20.7)	29.8 ± 3.3	14.7 ± 5.6	9.4 ± 4.4	32.7 ± 2.6	17.2 ± 3.0
	diploma	25(32.3)	30.0 ± 6.5	17.6 ± 4.8	9.4 ± 3.9	33.1 ± 3.1	16.5 ± 5.3
	Colleges	36(47.0)	31.2 ± 3.8	18.7 ± 18.0	10.6 ± 3.6	32.3 ± 2.6	16.3 ± 3.4
Job	Retired	51(66.2)	31.4 ± 3.6 *	18.9 ± 15.2	10.4 ± 3.8	32.6 ± 2.5	16.2 ± 3.7
	Unemployed	26(33.8)	28.8 ± 6.1	14.8 ± 5.5	9.4 ± 3.9	32.8 ± 2.9	17.2 ± 4.6
History of falling	Yes	21(27.3)	30.2 ± 5.3	21.8 ± 23.0 *	10.2 ± 4.1	32.2 ±2.2	16.9 ± 2.5
	No	56(72.7)	30.7 ± 4.6	15.9 ± 5.1	10.0 ± 3.8	32.8 ± 2.8	16.4 ± 4.4
Disease	0	10(13.0)	31.1 ± 4.2 *	28.22 ± 32.89 *	11.3 ± 5.1	32.5 ± 2.6	17.5 ± 4.9
	1	39(50.6)	31.6 ± 3.5	16.1 ± 4.8	10.2 ± 3.4	33.0 ± 2.7	17.0 ± 3.0
	≥2	28(36.4)	28.8 ± 5.9	15.7 ± 5.0	9.4 ± 4.0	32.26 ± 2.6	15.6 ± 4.7
Diabetes	Yes	17(22.1)	30.0 + 5.5	15.3 + 4.6	10.0 + 3.8	32.1 + 2.9	14.9 + 5.2 *
	No	60(77.9)	30.7 + 4.5	18.1 + 14.3	10.1 + 3.9	32.8 + 2.6	17.0 + 3.5
Blood Pressure	Yes	24(31.2)	29.4 ± 5.0	16.6 ± 4.4	10.2 ± 3.7	32.7 ± 2.9	15.6 ± 4.8
	No	53(68.8)	31.0 ± 4.6	17.9 ± 15.2	10.0 ± 3.9	32.6 ± 2.6	17.0 ± 3.5
Hyperlipidemia	Yes	12(15.6)	32.1 ± 3.6	17.0 ± 6.6	12.1 ± 4.0 *	33.3 ± 2.0	17.4 ± 3.1
	No	65(84.4)	30.2 ± 4.9	17.6 ± 13.7	9.7 ± 3.7	32.5 ± 2.8	16.4 ± 4.1
Medication Use	0–2	47(61.0)	31.2 ± 3.6	18.6 ± 15.9	10.3 ± 3.9	32.6 ± 2.2	16.7 ± 3.3
	≥3	30(39.0)	29.4 ± 6.1	15.8 ± 5.2	9.8 ± 3.7	32.8 ± 3.3	16.3 ± 4.9

* *p* < 0.05.

**Table 2:** Correlations (as Pearson r correlation coefficients) between balance, working memory, executive function, sustained attention, divided attention, and domains of retained activity participation of community-dwelling older adults.

Variables	Instrumental Activity	Social Activity	Low-Effort Leisure Activity	High-Effort Leisure Activity
Balance	0.2 *	0.1	0.01	0.3 *
(N-back) (n/s)	0.1	0.09	0.05 *	0.1 *
(Wisconsin Card Sort) (n/s)	0.01	0.1 *	0.07	0.03
(Sustained attention) (n/s)	0.08	0.03	0.04	0.03
(Divided attention) (n/s)	0.1	0.1	0.006	0.2 *

* *p* < 0.05.

**Table 3:** The univariate general linear model analysis for the relationship between demographic characteristics, health status, and domains of retained activity participation with sustained attention and divided attention in older adults.

Variable		Sustained Attention ^a^					Divided Attention ^b^				
		*β*	Lower	Upper	Sig	Multiplicity Adjusted *p*-Value	Β	Lower	Upper	Sig	Multidiciplinary Adjusted *p*-Value
Age (Year)		−0.08	−0.2	0.07	0.2	0.8	−0.0	−0.2	0.2	0.7	1
Gender	Male	−1.0	−2.7	0.5	0.2	0.8	0.7	−1.6	3.1	0.5	1
	Female	R **	.	.	.	.	R	.	.	.	.
Education level	Elementary–Middle	−0.8	−1.3	3.0	0.4	1	−0.8 *	2.2	14.0	0.02	0.08
	diploma	−1.0	−0.7	2.8	0.2	0.8	−0.5	−2.0	3.1	0.6	1
	Colleges	R	.	.	.	.	R	.	.	.	.
Job	Retired	1.1	−0.8	3.2	0.2	0.8	0.4	−3.3	2.5	0.7	1
	Unemployed	R	.	.	.	.	R	.	.	.	.
History of falling	Yes	−0.1	−1.7	1.4	0.8	1	−0.5	−1.7	2.8	0.6	1
	No	R	.	.	.	.	R	.	.	.	.
Number of Disease	0	1.4	−1.4	4.2	0.3	1	0.9	−3.1	5.1	0.6	1
	1	1.8 *	0.1	3.8	0.04	0.1	−1.4	−1.4	4.3	0.3	1
	≥ 2	R	.	.	.	.	R	.	.	.	.
Hypertension	Yes	−0.6	−1.1	2.3	0.4	1	−0.5	−3.0	2.0	0.6	1
	No	R	.	.	.	.	R	.	.	.	.
Diabetes	Yes	−0.4	−2.2	1.3	0.6	1	−1.6	−4.2	0.9	0.2	0.8
	No	R	.	.	.	.	R	.	.	.	.
Hyperlipidemia	Yes	−0.9	−1.1	3.0	0.3	1	−2.3 *	0.6	5.3	0.04	0.1
	No	R	.	.	.	.	R	.	.	.	.
Medication Use	0–2	−0.6	−2.3	1.3	0.5	1	−0.5	−3.2	2.2	0.7	1
	≥3	R	.	.	.	.	R	.	.	.	.
Types of activity	Instrumental activity	0.01	−0.05	0.08	0.7	1	0.02	−0.1	0.07	0.6	1
	High-effort leisure activity	0.001	−0.04	0.05	0.9	1	0.05 *	0.09	0.01	0.04	0.1
	Low-effort leisure activity	0.08 *	0.03	0. 1	0.04	0.1	0.02	−0.04	0.09	0.45	0.1
	Social activity	0.02	−0.1	0.04	0.46	1	0.08	−0.19	0.02	0.1	0.4

* *p* < 0.05, ** R = Reference, ^a^ R^2^ = 0.11, ^b^ R^2^ = 0.18.

**Table 4:** Univariate linear regression analysis for the relationship between demographic characteristics, health statuses, and domains of retained activity participation with balance, executive function, and working memory.

		Balance ^a^					Executive Function ^b^					Working Memory ^c^				
Variable		Β	Lower	Upper	Sig	Multiplicity Adjusted *p*-Value	*β*	Lower	Upper	Sig	Multiplicity Adjusted *p*-Value	*β*	Lower	Upper	Sig	Multiplicity Adjusted *p*-Value
Age (Year)		−0.1 *	0.07	0.3	0.03	0.1	−0.6	−0.5	0.1	0.1	0.4	−2.9	−1.0	0.3	0.4	1
Gender	Male	0.9	−1.4	3.4	0.4	1	−1.1 *	0.4	8.0	0.04	0.1	2.8	−4.3	10.1	0.4	1
	Female	R **	.	.	.	.	R	.	.	.	.	R	.	.	.	.
Education level	Elementary–Middle	−0.2	−2.9	3.4	0.8	1	−1.6	−4.6	1.3	0.2	0.8	−1.0	−10.5	8.4	0.8	1
	diploma	−0.1	−2.5	2.7	0.9	1	−0.08	−2.5	2.4	0.9	1	−1.5 *	6.3	15.5	0.04	0.1
	Colleges	R	.	.	.	.	R	.	.	.	.	R	.	.	.	.
Job	Retired	3.3 *	0.3	6.4	0.03	0.1	1.2	−1.5	2.4	0.9	1	4.8 *	1.1	13.8	0.02	0.08
	Unemployed	R	.	.	.	.	R	.	.	.	.	R	.	.	.	.
History of falling	Yes	−0.5	−2.9	1.8	0.6	1	−0.9	−1.3	3.1	0.4	0.1	−5.7	−1.2	12.8	0.1	0.4
	No	R	.	.	.	.	R	.	.	.	.	R	.	.	.	.
Number of Disease	0	3.9 *	0.2	8.2	0.04	0.1	5.1 *	1.1	9.1	0.01	0.04	10.5 *	2.0	23.0	0.04	0.1
	1	2.8 *	0.0	5.8	0.04	0.1	2.1 *	0.5	11.9	0.01	0.04	0.8	−9.4	7.7	0.8	1
	≥2	R	.	.	.	.	R	.	.	.	.	R	.	.	.	.
Hypertension	Yes	−1.6	−1.2	0.9	0.2	0.8	−1.5	−0.8	3.9	0.2	0.8	−2.2	−5.3	9.8	0.5	1
	No	R	.	.	.	.	R	.	.	.	.	R	.	.	.	.
Diabetes	Yes	−0.4	−3.1	2.2	0.7	1	−0.03 *	2.5	6.4	0.04	0.1	−1.9 *	6.7	9.9	0.04	0.1
	No	R	.	.	.	.	R	.	.	.	.	R	.	.	.	.
Hyperlipidemia	Yes	−2.1	−0.8	5.2	0.1	0.4	−3.6 *	0.7	6.5	0.01	0.04	−1.0	−8.0	10.0	0.8	1
	No	R	.	.	.	.	R	.	.	.	.	R	.	.	.	.
Medication Use	0–2	−1.3	−4.1	1.5	0.3	1	−0.05	−2.5	2.62	0.9	1	−0.7	−7.6	9.0	0.8	1
	≥3	R	.	.	.	.	R	.	.	.	.	R	.	.	.	.
Types of activity	Instrumental activity	0.01	−0.8	0.1	0.7	1	0.03	−0.1	0.05	0.4	1	0.07 *	0.1	7.4	0.04	0.1
	High-effort leisure activity	0.1 *	0.04	0.1	0.001	0.004	0.02 *	0.03	0.1	0.04	0.1	0.1	−0.3	0.03	0.1	0.4
	Low-effort leisure activity	0.02	−0.09	0.04	0.4	1	0.04	−0.02	0.1	0.2	0.8	0.1	−0.04	0.3	0.1	0.4
	Social activity	0.008	−0.1	0.1	0.8	1	0.02	−0.08	0.1	0.6	1	0.2	−0.1	0.5	0.1	0.4

* *p* < 0.0, ** R= Reference, ^a^ R^2^ = 0.45, ^b^ R^2^ = 0.13, ^c^ R^2^ = 0.25.The univariate general linear model analysis results showed associations between retained activity participation and background-health variables with balance, executive function, and working memory, and are presented in Table 4. As expected, the result shows that men scorer 1.1times lower in executive function compared to women (*p* < 0.05). Thirteen percent (13%) of the executive function is explained by retained activity participants and the demographic variables. In addition, those who were employed in the past and are retired now increase their balance and working memory by 3.3 and 4.8 compared to those who were without work (including household) (*p* < 0.05). Moreover, the univariate general linear model analysis showed that the number of diseases was inversely associated with balance (*β* = 3.9, *p* < 0.05), executive function (*β* = 2.3, *p* < 0.05) and working memory (*β* = 10.5, *p* < 0.05). Furthermore, we found that a 1 unit increase in high-effort leisure activity can increase the balance score by 0.1 and increase the executive function score by 0.02; a 1 unit increase in instrumental activity can increase the working memory score by 0.07 (*p* < 0.05). The R^2^ of the working memory and balance test were 0.25 and 0.45, respectively. In other words, 25% of the working memory and 45% of the balance are explained by retained activity participants and the demographic variables. Next, statistically significant independent variables identified in the univariate general linear model were entered into a multivariable general linear model. Finally, the multivariate regression analysis results indicated that individuals with high-effort leisure activity were more likely to have better cognition and balance (Wilks’ Lambda value = 0.7, F = 4.8, *p* < 0.001).

## Data Availability

Data can be made available upon request to the corresponding authors.
